# Extracellular Vesicles as Biomarkers in Infectious Diseases

**DOI:** 10.3390/biology14020182

**Published:** 2025-02-11

**Authors:** Cinthia Gonzalez Cruz, Husain M. Sodawalla, Thalachallour Mohanakumar, Sandhya Bansal

**Affiliations:** 1 Barrow Neurological Institute, St. Joseph’s Hospital and Medical Center, Phoenix, AZ 85013, USA; cinthia.gonzalezcruz@commonspirit.org; 2 Department of Mechanical Engineering, Northern Arizona University, Flagstaff, AZ 86011, USA; hs628@nau.edu; 3 Norton Thoracic Institute, St. Joseph’s Hospital and Medical Center, Phoenix, AZ 85013, USA; tm.kumar@commonspirit.org

**Keywords:** extracellular vesicles, biomarkers, disease detection

## Abstract

Extracellular vesicles (EVs) are nanosized vesicles that are secreted by all cells into the extracellular space. EVs are involved in cell-to-cell communication and can be found in different bodily fluids (bronchoalveolar lavage fluid, sputum, and urine), tissues, and in circulation; the composition of EVs reflects the physiological condition of the releasing cell. The ability to use EVs from bodily fluids for minimally invasive detection to monitor diseases makes them an attractive target. EVs carry a snapshot of the releasing cell’s internal state, and they can serve as powerful biomarkers for diagnosing diseases. EVs also play a role in the body’s immune and pathogen detection responses. Pathogens, such as bacteria and viruses, can exploit EVs to enhance their survival and spread and to evade detection by the immune system. Changes in the number or contents of EVs can signal the presence of an infection, offering a potential avenue for developing new diagnostic methods for infectious diseases. Ongoing research in this area aims to address current challenges and the potential of EVs as biomarkers in diagnosing a range of diseases, including infections and infectious diseases. There is limited literature on the development of EVs as diagnostic biomarkers for infectious diseases using existing molecular biology approaches. We aim to address this gap by reviewing recent EV-related investigations in infectious disease studies.

## Simple Summary

Extracellular Vesicles (EVs) hold immense potential as non-invasive biomarkers and drug-delivery vehicles for infectious diseases. Secreted by most human cells, EVs carry specific DNA, RNA, proteins, and metabolites, with their contents reflecting the individual’s clinical condition and cellular state. This makes them valuable tools for understanding disease mechanisms. EVs are being extensively explored as biomarkers in complex diseases, including various infections. There is a connection between EVs and pathogen-host interactions. However, significant gaps remain in understanding the roles of EVs in infection, pathogenesis, and related immune mechanisms. Addressing these gaps is crucial for advancing diagnostic and therapeutic strategies. In this article, we have examined how EVs can serve as diagnostic biomarkers in infectious diseases. These advances pave the way for EVs as biomarkers, highlighting the importance of EVs in the future of diagnostics and precision medicine.

## 1. Introduction

Infectious diseases caused by bacteria, viruses, fungi, parasites, and prions pose a significant global health challenge, accounting for approximately 13.7 million deaths annually [1,2,3]. Their timely and accurate diagnosis is crucial for instituting targeted therapies. Extracellular vesicles (EVs) released by a host’s immune cells during an infection carry a diverse cargo, including small molecules, proteins, nucleic acids (i.e., RNA and DNA), and metabolites [4,5], and can be detected in bodily fluids through non-invasive or minimally invasive methods. Thus, EVs show promise as an emerging diagnostics tool in the arsenal against infectious diseases [6].

Up until the 1980s, EVs were thought of as ‘cell’s garbage bins’ instrumental in removing unnecessary proteins and biological substances from cells during cell maturation processes [7]. Current literature shows cells release EVs for normal physiological functions like intercellular communications and cellular proliferation [8]. EVs can also be released under pathological conditions to initiate a niche tumor site [9] or facilitate a viral condition [10]. The biomolecules encapsulated within a released EV may have downstream functional implications in activating immune responses through direct antigen presentation or endocytosis, but the exact mechanisms are currently under active investigation [11,12].

Research in elucidating the role of extracellular vesicles in cancer disease progression and diagnostic biomarker study has made several strides but their role in infectious disease space is underexplored. There is an urgent and unmet need for developing noninvasive, time-efficient, and cost-effective EV-based diagnostic methods for infectious diseases. This review will thus focus on explaining the role of EVs as a diagnostic biomarker tool in infectious diseases split across three main pathogen types: bacterial, fungal, and viral infections [13,14].

## 2. Extracellular Vesicles

**Figure 1:**
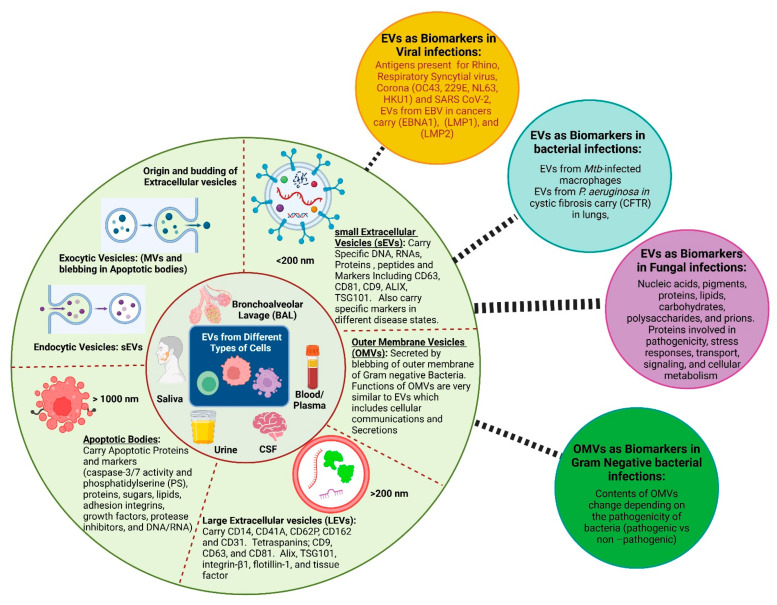
Extracellular vesicles: sources, origin, and biomarkers in infectious diseases (Created with BioRender.com).

According to the recent Minimal Information for Studies of Extracellular Vesicles (MISEV 2023)—a report generated by the International Society for Extracellular Vesicles (ISEV) ISEV, extracellular vesicles are lipid bilayer membrane-delimited particles that can be categorized as small (sEVs; <200-nm in diameter), large (lEVs; >200-nm in diameter), or apoptotic bodies (>1000-nm in diameter) [15,16]. Using the terms exosomes and microvesicles has been discouraged until there is a discussion of subcellular origin [17]. EVs are shown to carry a variety of biological molecules including proteins and peptides, lipids, nucleic acids, small metabolites, and antigens to infectious agents [18,19]. The composition of EVs depends on their cellular origin and the functional state of the cell [20] (Figure 1). 

## 3. Source and Release of Extracellular Vesicles

sEVs can be formed through the inward budding of the endosomal membrane by the endosomal sorting complex (ESCRT) that forms multivesicular bodies within the cell [21]. These multivesicular bodies fuse with the plasma membrane, releasing the sEVs from the cell. sEVs are released by cells through endocytosis. Generally, the metabolic state of the cells releasing EVs determines the types and amounts of molecules present in them. However, certain biomarkers CD9, CD63, CD81, tetraspanins, TSG101, and syntenin [17,22] are constitutively present on endosomal sEVs. The release and contents of sEVs are influenced by the physiological and pathological circumstances of the cells [23,24,25]. Thus, an interaction of host and pathogen cells during an infection can dictate the presence of biological contents in the sEVs [26].

Conversely, large EVs (lEVs) are shed directly from the outward budding of the plasma membrane. This process involves the direct protrusion and pinching off of membrane segments [27]. The plasma membrane undergoes localized protrusion and budding. The protrusions pinch off from the cell surface, forming microvesicles [27,28].

Apoptotic bodies are formed during the programmed cell death (apoptosis). As cells undergo apoptosis, they fragment into membrane-bound bodies containing cellular debris, including organelles and nuclear fragments. The apoptotic cell undergoes shrinkage and membrane blebbing [29]. These blebs and fragments break off, forming apoptotic bodies containing cellular debris. They help in the safe disposal of cellular remnants and may contain proteins, lipids, and nucleic acids that can influence surrounding cells or trigger immune responses [30,31]. Diseased cells secrete EVs that facilitate or suppress immune responses and enable intricate communication between immune cells [32]. Fragmented double-stranded DNA can get integrated into the genome of healthy cells through EV-mediated transfer followed by activation of the DNA damage repair pathway and apoptosis [33,34].

In gram-negative bacterial species, outer membrane vesicles (OMVs) are secreted by blebbing of the outer membrane [35,36,37]. The functions of OMVs are very similar to EVs and include cellular communications and secretions. The contents of the OMVs change depending on the pathogenicity of bacteria (pathogenic vs. nonpathogenic). This area of research has recently gained much attention but is still an underexplored area with limited published research [35,36,37,38].

## 4. Extracellular Vesicles as Biomarkers in Infectious Diseases

Infectious diseases remain a predominant cause of global morbidity and mortality, and this burden is particularly pronounced in low- and middle-income countries, where factors such as limited access to effective treatments and underdeveloped healthcare infrastructure exacerbate their impact [39]. Infections can vary from aggressive and life-threatening to non-hostile, relatively mild, short-term or chronic and can spread from an infected person, animal, or contaminated object to a susceptible host [1,40,41]. One promising area of investigation in infectious disease management is the study of EVs. EVs are of significant interest in infectious disease research due to their involvement in cell-to-cell communication and their capacity to modulate immune responses [42]. It has been widely reported that parasitic organisms, including bacteria, viruses, fungi, and parasites, release EVs during [43] lower respiratory tract infections, tuberculosis, gastrointestinal diseases, sexually transmitted diseases, HIV/AIDS, skin diseases, malaria, SARS-CoV-2 [44], and zoonotic diseases [45].

EVs can be either directly infectious [46] or mediate toxic reactions via altering the cellular contents. EVs can be both host- and pathogen-derived based on a given host-pathogen interaction. They can mediate infections via transferring pathogen-related molecules and, in some cases, an entire pathogen [47]. EVs can cause healthy cells to become more susceptible to infection by transferring viral components, including proteins and genomic molecules, from infected cells. The cargos that EVs carry provide insights into cellular processes and disease states and may be useful as noninvasive biomarkers. EVs have been successfully isolated from various bodily fluids, including blood, urine, saliva, bronchoalveolar lavage fluid, and cerebrospinal fluid. The amount and size of EVs in each biological sample can be measured using Nanosight (NS300, (Malvern Panalytical, Malvern, UK)), a nanoparticle tracking analyzer, and ExoviewR200, (NanoView Biosciences now Unchained Labs, Pleasanton, CA, USA) instruments. EVs can be measured before or after isolation and purification by using standardized methods i.e., kit-based methods, ultracentrifugation, and sucrose gradient purification [48,49,50,51,52].

**Figure 2:**
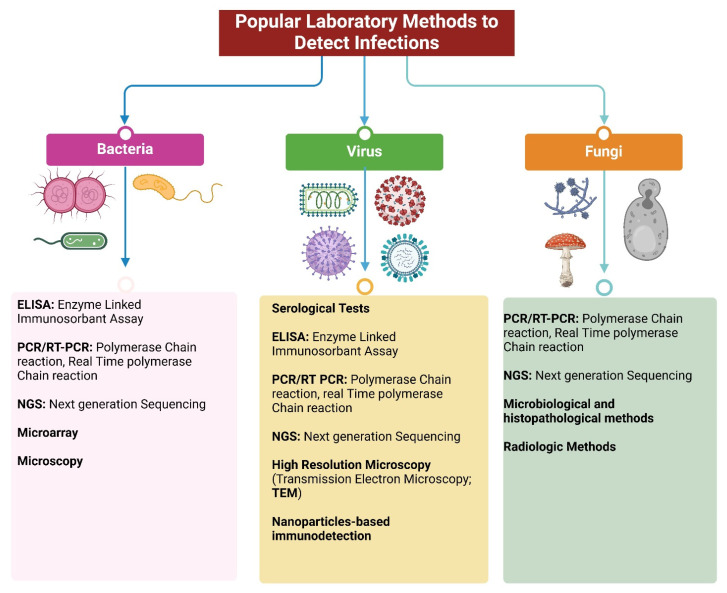
Molecular biology-based laboratory methods to detect infections: bacterial, viral, or fungal (Created with BioRender.com).

Current diagnostic laboratory-based methods focus on analyzing host specimens for evidence of the infectious agent or evidence of immunity to an agent. These methods include polymerase chain reaction (PCR), enzyme-linked immunosorbent assay (ELISA), pulsed-field gel electrophoresis, next generation sequencing, and microarray analysis [53,54,55,56,57,58,59] (Figure 2). Laboratory-based ELISAs can detect antibodies produced in response to infections (bacterial, viral) and can indicate a prior or ongoing infection depending on subclasses of antibodies (IgG, IgM, IgA) [53,54,58]. PCR can amplify pathogenic DNA or RNA from patient samples to identify specific pathogens rapidly and accurately, providing specific and sensitive identification of many bacterial, viral, and fungal infections. Real-time PCR can also quantify viral load and detect viral presence in real time [55].

Next generation sequencing is an advanced high-throughput laboratory technique to identify DNA or RNA, providing comprehensive microbial profiling for complex infections. Gene and protein microarrays can detect multiple bacterial pathogens simultaneously from a single sample using a DNA or RNA chip. Although these laboratory assays are very advanced, they not only need specialized instruments but also a dedicated facility to avoid external contamination by pathogens. These molecular biology techniques primarily focus on detecting infectious agents during active infections. While these approaches are valuable, there is an ongoing demand for new tools that offer greater efficiency and cost-effectiveness in pathogen detection and disease monitoring.

EVs present a promising avenue for achieving these goals. Our research has demonstrated that EVs from patients suffering from chronic lung diseases (e.g., Chronic Lung Allograft Dysfunction or CLAD) and infections (Respiratory Syncytial Virus, Rhino, Corona, and SARS-CoV2 Viral Infections), have the potential to predict disease severity well in advance of its clinical onset [60]. This finding underscores the predictive power of EVs in identifying early pathological changes, which could revolutionize disease prognosis and management. In addition to our work, other research groups have highlighted the potential of EV proteomics to identify disease-specific biomarkers [34]. These studies showcase the versatility of EVs in serving as a rich source of biological molecules, both within their cargo and on their surface, which can be leveraged to develop precise and disease-specific biomarkers. By utilizing the inherent biological information carried by EVs, molecular biology tools can be enhanced to provide sensitive, specific, and cost-effective diagnostic solutions. This could pave the way for early detection and personalized interventions across a range of infectious and non-infectious diseases.

The contents of EVs change in response to pathological conditions, making them useful for detecting and monitoring diseases. For example, changes in miRNA, protein, and metabolic profiles in EVs can reflect the disease state of the cells such as infectious disease, lung diseases, cancer, cardiovascular diseases, and neurological disorders. EVs can potentially provide early signals of disease before symptoms arise or before traditional biomarkers become detectable [61,62]. They also offer a means to monitor disease progression and response to treatment in real time [63,64]. Since EVs can be extracted without tissue biopsies and rapidly assayed for biomarker levels within their membranes, they can be readily harnessed towards dynamic monitoring of ongoing infection.

### 4.1. Extracellular Vesicles as Biomarkers in Bacterial Infections

Bacterial EVs are spheroidal structures with a lipid bilayer, ranging from 100 to 500 nm in diameter are reported to contain various biomolecules such as proteins, DNA, and RNA [65]. Specifically, outer-membrane vesicles from gram-negative bacteria play a crucial role in communication with nearby bacteria, the surrounding environment, and the host [66]. Outer-membrane vesicles are continuously produced throughout the bacteria’s life cycle and are involved in trafficking virulence factors directly into host cells [67]. These vesicles enter host cells via phagocytosis, endocytosis, or membrane fusion, enabling the bacteria to influence host genome activity [68]. This interaction can trigger toll-like receptor (TLR) responses [69], leading to the release of pro-inflammatory and immunomodulatory cytokines, while also mediating liposaccharide (LPS) tolerance by suppressing other proinflammatory cytokines such as tumor necrosis factor-alpha (TNF-α) [70]. Research into outer-membrane, vesicle-mediated host-pathogen interactions is crucial for deepening our understanding of bacterial pathogenesis and immune modulation and may lead to novel therapeutic approaches for treating chronic, antibiotic-resistant bacterial infections.

Despite the availability of a vaccine, tuberculosis (TB) remains a significant public health challenge, claiming 1.5 million lives annually, partly due to complications arising from drug resistance [71]. *M. tuberculosis* primarily infects alveolar macrophages, altering the immune environment by recruiting additional immune cells to the infection site, thereby modulating the immune response and evading the host’s immune system [72]. EVs secreted by *M. tuberculosis*-infected macrophages can disseminate the infection to previously uninfected macrophages [73]. In a study by Walters et al., cultured, bone marrow-derived macrophages were infected with *M. tuberculosis*. The EVs secreted from these cultures were then injected into uninfected mice, leading to a significant recruitment of innate immune cells to the injection site [73]. Javadi A et al. found that when they mixed higher concentrations of serum EVs (derived from tuberculosis patients) with THP1 cells, it led to an overexpression of apoptotic miRNAs and increased cell death. [74]. This indicates that the EV-miRNA profile of a TB-infected patient could be utilized as a biomarker tool for active tuberculosis infections, offering the possibility of a quicker diagnosis and paving the way for future screening of drug-resistant tuberculosis.

*Pseudomonas aeruginosa* is another gram-negative bacteria associated with multidrug-resistant infections leading to significant mortality [75]. This opportunistic pathogen is capable of colonizing various human environments such as the throat, skin, lungs, and gastrointestinal tract [76]. Although *P. aeruginosa* thrives in diverse settings like water and soil, infections predominantly occur in healthcare environments, particularly on ventilators, catheters, and surgical wounds [77]. The role of EVs in *P. aeruginosa* pathogenicity has been well-documented, particularly regarding the budding of membrane vesicles and their internal cargo [78]. The cystic fibrosis transmembrane conductance regulator (CFTR), essential for maintaining salt and water homeostasis in the lungs, is inhibited by the CFTR inhibitory factor protein secreted by *P. aeruginosa*, facilitating its colonization in the lungs [79]. Bauman and Kuehn conducted a study in which they cultured a laboratory strain of *P. aeruginosa* and a strain isolated from a patient with cystic fibrosis to quantify, purify, and characterize the secreted microvesicles. Their findings revealed that the cystic fibrosis-isolated strain had a higher abundance of PaAP (PA2939), an aminopeptidase linked to biofilm formation and nutrient acquisition from complex sources [80]. This enzyme is advantageous for the bacterial community, enhancing cell-to-cell communication and cooperative behavior, thereby increasing individual fitness [81]. Understanding these specific characteristics of *P. aeruginosa* EVs and their contribution to virulence factors has significant implications for developing targeted therapies. Disrupting this cell-to-cell communication system could reduce the fitness of pathogenic *P. aeruginosa*, potentially decreasing its drug resistance and lowering mortality.

### 4.2. Extracellular Vesicles as Biomarkers in Fungal Infections

Fungal infections can cause severe disease and cause over 1.5 million deaths worldwide annually [82]. Individuals affected by immunocompromising/immunodeficiency disease (e.g., HIV/AIDS) are a high-risk group for serious fungal infections [83,84]. Although fungal EVs reportedly carry an abundance of pathogenic proteins and signaling molecules related to other physiological processes, there is a gap in knowledge and mechanistic understanding of EVs and fungal infections. According to some reports, virulence factors can be transported via EVs from fungal cells [85].

The first fungal phytopathogen ever reported to produce EVs was the filamentous, environmental fungus *Alternaria infectoria* [86]. In recent years, EVs derived from fungi have gained attention as researchers have revealed that fungal EVs carry a range of molecules that are capable of modulating host immune response [87]. Fungal EVs can positively modulate immunity activation, e.g., EVs from *Candida albicans* show immunomodulatory effects eventually activating innate immune responses.

Vargas et al. have shown in an in vivo experiment that when immunosuppressed mice were vaccinated with EVs and Freund’s adjuvant, levels of inflammatory cytokine biomarkers like TNF-α, IL-12p70, and IFN-γ increased [88]. With the emergence of new fungal pathogens and resistant strains as serious threats to global health, EVs may offer a new strategy against fungal diseases due to their ability to modulate the immune system and transfer bioactive components [88,89,90].

### 4.3. Extracellular Vesicles as Biomarkers in Viral Infections

Since EVs and viruses have many structural similarities in their size, structure, generation, and uptake [91] and even their modes of reaching the recipient cells are the same. Accumulating evidence demonstrates that pathways to formation of host EVs are hijacked by viruses, which leads to the formation of virally modified exosomes, further contributing to virus spread and immune evasion [92,93].

Cargo loading of EVs may be affected by viral infections, and this altered loading can in turn alter the immune response of the host. Very little is known about the diversity of pathogen-derived nucleic acids, proteins, and lipids in EVs. Previous assumptions about cargo loading of EVs during viral infections have been based on the understanding of viral packaging and propagation [94,95]. Meckes and Raab-Traub postulated that EV and viruses mediate the intercellular transfer of functional cellular proteins, mRNAs, and miRNAs in a similar fashion [96]. Izquierdo-Users et al. revealed that after fusing with dendritic cells, a sorting of HIV-1 particles and antigens occurs in EV-like vesicles [97]. Van Dongen et al. have shown that EVs can activate viral infections by carrying and transferring viral antigens to CD4 T cells [98].

#### 4.3.1. Extracellular Vesicles as Biomarkers in Common Viral Infections

Viruses like herpesvirus, sarcoma-associated virus, and Epstein-Barr virus (EBV) have been studied to untangle their complex interactions with the host’s immune system in contraction and progression of their diseased state [99,100]. EVs isolated from EBV-infected cells contain the enzyme dUTPase, which further activates transcription factor NFkB and also promotes cytokine release from primary dendritic cells and peripheral blood mononuclear cells, leading to a proinflammatory antiviral response via CXCL11 [97,101]. It is postulated that when cytomegalovirus (CMV) infects human endothelial cells, EVs that can stimulate memory CD4^+^ T cells are released through the transfer of antigens to allogeneic dendritic cells [102]. Vesicles released by CMV-infected human cells contain soluble molecules that belong to the lectin family and are present in CMV glycoprotein B, which makes recipient cells more susceptible to CMV infection [103]

Viruses exploit EVs by manipulating their cargo to incorporate viral RNA, DNA, and proteins, effectively hijacking EVs to facilitate viral spread [104]. Non-enveloped viruses, such as norovirus, poliovirus, and hepatitis A, can be shed as individual viral particles or as clusters within vesicles [105]. This encapsulation allows for the delivery of a higher infectious dose, enhancing the likelihood of a successful infection [106]. These vesicle-encased viral clusters have demonstrated resilience against temperature variations, detergents, and UV radiation, a resilience attributed to the high concentration of viral particles within the vesicles [107].

Although the treatment and prevention of HIV/AIDS has improved tremendously, it remains a significant global public health concern. Over 42 million people have died from HIV/AIDS since the beginning of the epidemic, with an alarming 630,000 people in 2022, despite commercially available therapies [108]. HIV-1 infects immune cells and depletes helper CD4^+^ T cells [109]. EVs play an ambiguous role in HIV infection as shown by Dias MVS et al. [110] inflicting both pro- and anti-viral effects. EVs from HIV-infected patients can transport host-derived restriction factors to nearby cells and thus trigger antiviral responses [111]. Antiviral effects include the presence of CD4 on the surface of EVs released by CD4 T-cells, which can potentially impair HIV-1 by acting as a decoy for CD4 T-cells and neutralizing HIV-1 virions, thereby preventing the spread of the virus [112].

The hepatitis C virus (HCV), a member of the Flaviviridae family, is a human virus with a strong tropism for the liver and one of the leading causes of liver damage, causing chronic hepatitis in about 80% of infected individuals. The virus induces pathogenesis primarily through disruptions in cytokines, chemokines, and growth factors, promoting extracellular matrix production and reducing its degradation via matrix metalloproteinases. These processes contribute to liver fibrosis, which can progress to cirrhosis and, in some cases, hepatocellular carcinoma [113]. However, during HCV infection, the cargo of EVs is not limited to viral components. In fact, cytokines and other factors that facilitate viral replication have also been found in EVs.

Zika virus, a mosquito-borne flavivirus, poses a public health concern in certain regions of the world [114]. Although mostly asymptomatic, Zika virus infection can lead to serious neurological complications in some cases [115,116]. Additionally, Zika virus infection can trigger antibody-dependent enhancement in infections with related viruses. EVs isolated from Zika-infected cells do not directly transmit viral infections, but they do carry high levels of envelope proteins on their surface. These envelope proteins are crucial because they present a complex antigenic landscape that can interact with the immune system [117]. Zika virus specifically modifies the density, cargo, and secretion of EVs, which contain viral RNA that is infectious [118].

Most research on viral EVs has focused on HIV-1 and EBV, but significant findings have also been made for other viruses, including SARS-CoV-2. Limited study has been done on EVs in hepatitis C virus and the Zika virus which can play a role in the dissemination of viral RNA and proteins to neighboring cells and in promoting infection [119]. Although the exact mechanisms and roles of EVs in various viral infections and infectious diseases are still being studied, our understanding of how they interact with the immune system whether by activating or suppressing it—remains limited.

#### 4.3.2. Extracellular Vesicles as Biomarkers in Viral Infections in Solid Organ Transplant

Solid organ transplantation is the final option for patients with end-stage organ failure (lungs, heart, kidney, etc.). EVs possess the potential to serve as biomarkers in lung transplant recipients, but it is not limited to only lung transplants. Rejection is a common problem after any solid organ transplant [120,121]. Major diseases which contribute to advanced lung failure include cystic fibrosis, idiopathic pulmonary fibrosis, chronic obstructive pulmonary disease, autoimmune disease, and respiratory infections [122,123], but the underlying mechanisms of these disease states that lead to lung failure are not clearly understood. Our group has demonstrated the presence of specific antibodies, antigens, and proteins in EVs as biomarkers in solid organ transplant recipients [22,49,50,124,125].

We have demonstrated the presence of viral antigens on EVs during respiratory viral infections in lung transplant recipients. Patients with respiratory viral infections such as rhino, respiratory syncytial virus, and corona viruses including SARS-CoV-2 release EVs that carry viral antigens [48,126]. In a 2021 study, we have reported the release of EVs with SARS-CoV-2 antigens even before the development of antibodies after immunization [51]. In an ongoing study, Examination of the physiological impact of these viral antigens carrying EVs in lung transplant recipients could elucidate the underlying mechanisms associated with chronic allograft rejection.

Fleming et al. reported in 2014 that EVs released from infected cells contain virus particles that can infect healthy cells [127]. These EVs are also capable of altering the infected host’s immune responses. Our group is exploring the feasibility of EVs as a diagnostic tool in respiratory tract viral infections. A few groups [127,128], including our group [48,126,129], have already demonstrated that EVs play pivotal roles in SARS-CoV-2 infection as they carry the immunogenic spike antigen.

## 5. Challenges in Clinical Adoption of Extracellular Vesicles as Diagnostic Biomarkers

There has been a rapid advancement in the development of EVs as clinically relevant biomarkers. EVs can be used as biomarkers for diagnosing several lung diseases, cardiovascular diseases, immune disorders, cancers, bacterial infections, viral infections, fungal infections, and infectious diseases [130]. Infected human cells release EVs with specific antigens, RNAs, and DNA related to infectious metabolites which can be assayed as biomarkers [131].

Developing diagnostics from EVs is challenging due to variations in size and isolation methods. Yields and purity of EVs isolated from different bodily fluids are also a limiting factor for developing them as a diagnostic tool. Sensitive, sophisticated, and high-throughput techniques (mass spectroscopy, next generation sequencing, and multiomics techniques) are needed to define the molecular composition of EVs in different clinical conditions. Other limitations to adoption of EVs as a diagnostic tool include (a) lack of expertise to extract and process the EVs for serial monitoring, (b) lack of standard and optimized methods for isolation of sEVs specifically, (c) the complexity of analysis due to the nature of EV cargo requiring sophisticated analytical techniques and data interpretation, which is exacerbated due to the heterogeneity of EV populations, and finally, (d) extensive validation to translate the use of sEVs specificially into routine clinical assays.

There are multiple ongoing clinical trials to develop EVs as biomarkers. Ghodasara A. et al. discuss the details of ongoing clinical trials to develop EVs as biomarkers in different diseases in their report recently published in *Advanced Healthcare Materials* [132]. The clinical use of EVs is not limited to biomarkers, as EVs are also being developed as therapeutic agents, too [133].

## 6. Conclusions and Future Directions

In conclusion, this review highlights the critical, clinically relevant need for noninvasive, time-efficient, and cost-effective EV-based diagnostic methods for infectious diseases. Etiological research investigating the role of EVs in a given infection will help build the next generation of diagnostic tools with a higher predictive value. Considering that an infection-specific biomarker exists on a continuum based on the infection stage, the first challenge would be to improve and standardize the extracellular vesicle isolation techniques across clinical research labs. This will directly impact the sensitivity and specificity of a given EV-based infectious disease diagnostic tool.

Improved mechanobiological understanding of extracellular vesicles could help realize the promise of precision medicine. Literature shows that EVs could be harnessed as ‘nano-sized’ vehicles in therapeutic applications e.g., targeted drug delivery, gene therapy, and as part of vaccines [134]. By engineering EVs to carry specific therapeutic agents, it is possible to direct the agents precisely to targeted cells or tissues, potentially improving efficacy and reducing side effects.

## Abbreviations

CFTR	cystic fibrosis transmembrane conductance regulator
CMV	cytomegalovirus
EBV	Epstein-Barr virus
ELISA	enzyme-linked immunosorbent assay
ESCRT	endosomal sorting complex
EV	extracellular vesicle
mRNA	messenger RNA
miRNA	microRNA
OMV	outer membrane vesicle
PCR	polymerase chain reaction
sEV	small extracellular vesicle
